# Glucose-6-Phosphate Dehydrogenases: The Hidden Players of Plant Physiology

**DOI:** 10.3390/ijms232416128

**Published:** 2022-12-17

**Authors:** Zhengrong Jiang, Ming Wang, Michael Nicolas, Laurent Ogé, Maria-Dolores Pérez-Garcia, Laurent Crespel, Ganghua Li, Yanfeng Ding, José Le Gourrierec, Philippe Grappin, Soulaiman Sakr

**Affiliations:** 1 Institut Agro, University of Angers, INRAE, IRHS, SFR QUASAV, 49000 Angers, France; 2 College of Agronomy, Nanjing Agricultural University, Nanjing 210095, China; 3 Dryland-Technology Key Laboratory of Shandong Province, College of Agronomy, Qingdao Agricultural University, Qingdao 266109, China; 4 Plant Molecular Genetics Department, Centro Nacional de Biotecnología-CSIC, Campus Universidad Autónoma de Madrid, 28049 Madrid, Spain

**Keywords:** glucose-6-phosphate dehydrogenase, seed germination, apical dominance, sugar signaling, abiotic stress, ROS

## Abstract

Glucose-6-phosphate dehydrogenase (G6PDH) catalyzes a metabolic hub between glycolysis and the pentose phosphate pathway (PPP), which is the oxidation of glucose-6-phosphate (G6P) to 6-phosphogluconolactone concomitantly with the production of nicotinamide adenine dinucleotide phosphate (NADPH), a reducing power. It is considered to be the rate-limiting step that governs carbon flow through the oxidative pentose phosphate pathway (OPPP). The OPPP is the main supplier of reductant (NADPH) for several “reducing” biosynthetic reactions. Although it is involved in multiple physiological processes, current knowledge on its exact role and regulation is still piecemeal. The present review provides a concise and comprehensive picture of the diversity of plant G6PDHs and their role in seed germination, nitrogen assimilation, plant branching, and plant response to abiotic stress. This work will help define future research directions to improve our knowledge of G6PDHs in plant physiology and to integrate this hidden player in plant performance.

## 1. Introduction

**Figure 1:**
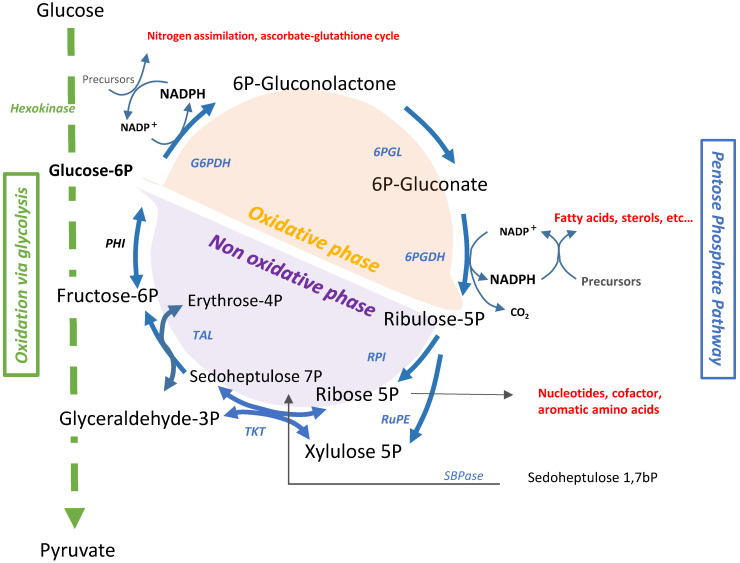
Overview of the reactions of the pentose phosphate pathway (PPP) and its connection to glycolysis. The glycolytic pathway is colored in green. The oxidative part of the PPP is colored in orange, the non-oxidative part in purple; one-headed arrows designate physiologically irreversible reactions, two-headed arrows reversible ones; abbreviation meanings: G6PDH, glucose-6-phosphate dehydrogenase (EC 1.1.1.49); 6PGL, 6-phosphogluconolactonase (EC 3.1.1.31); 6PGDH, 6-phosphogluconate dehydrogenase (EC 1.1.1.44); RPI, ribose-5-phosphate isomerase (EC 5.3.1.6); RuPE, ribulose-5-phosphate 3-epimerase (EC 5.1.3.1); TKT, transketolase (EC 2.2.1.1); TAL, transaldolase (EC 2.2.1.2); PHI, hexose-6-phosphate isomerase (EC 5.3.1.9); SBPase, sedoheptulose-1,7-bisphosphatase (EC 3.1.3.37); other details in the text.

Plant performance intrinsically depends on balanced carbon flux through interconnected metabolic hubs. Glucose-6-phosphate dehydrogenases (G6PDHs) catalyze a metabolic node between glycolysis and the pentose phosphate pathway (PPP), i.e., the oxidation of glucose -6-phosphate (G6P)—A substrate generated by hexokinase during glycolysis—to 6-phosphogluconolactone, concomitant with the production of the reductant nicotinamide adenine dinucleotide phosphate (NADPH). This reaction is considered as the rate-limiting step of the oxidative pentose phosphate pathway (OPPP), and controls OPPP-dependent carbohydrate allocation [1,2,3]. The PPP is divided into two branches with two distinct functions. The OPPP is the irreversible oxidative branch of the PPP. It comprises three irreversible reactions that convert glucose 6 phosphate (G6P) into carbon dioxide (CO_2_) and ribulose-5-phosphate (Ru5P, a pentose phosphate) while two molecules of NADPH are produced (Figure 1). Ru5P becomes the substrate of the reversible non-oxidative phase (NOPPP), which is the reversible branch of the PPP. The second branch is the non-oxidative pentose pathway (NOPP). It is composed of reversible transaldolase and transketolase reactions enabling the cell to control carbon flux between the PPP and glycolysis [3,4,5,6]. Including two NADPH-producing steps, the OPPP is the main provider of reductant (NADPH) for “reductive” biosynthetic reactions (e.g., assimilation of inorganic nitrogen, fatty acid synthesis) and for ROS scavenging. The NOPPP rather provides precursors (ribose-5-phosphate; erythrose-4-phosphate) for aromatic amino-acid, nucleotide, and cofactor synthesis [3,6]. The OPPP is an almost ubiquitous pathway, present in all eukaryotic cells and most bacteria, and seems to be more recent than the NOPPP in evolution [3,5].

G6PDH was discovered in the early 1930s, when Otto Warburg et al. investigated the enzymatic oxidation of glucose-6-phosphate to 6-phosphogluconate (6PG) in yeast. It was initially called *Zwischenferment* (ZWF1) or intermediate enzyme [5]. Plant G6PD-cDNA sequences were first isolated from potato [7,8,9] and have been identified in several monocot and dicot crops [4,10]. Although the available results are assigned to a variety of fundamental processes, including those related to plant growth and plant resistance to abiotic stress, most of them are still piecemeal. This situation hampers the building up of a comprehensive picture of its central role throughout plant life cycle and prevents us from obtaining further insights into the main regulatory network governing its involvement in plant growth and resistance to stresses. We propose a concise overview of the diversity of plant G6PDHs and their mechanisms of regulation, and of their role in four main plant physiological processes: seed germination, nitrogen assimilation, plant branching, and plant response to abiotic stresses. This work will provide a solid basis for future lines of research aimed at improving our knowledge of G6PDHs in plant physiology and integrating this hidden player in plant resilience to climate change.

## 2. Classification and Regulation of G6PDH

**Figure 2:**
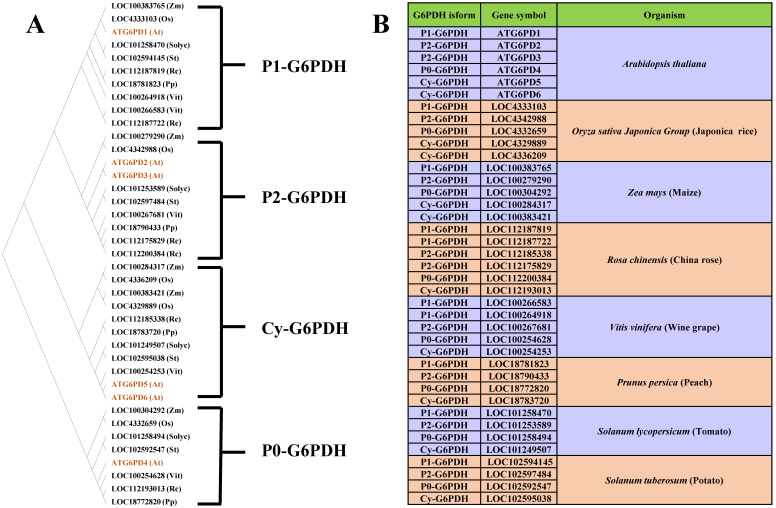
Comparison of different G6PDH isoforms from 8 higher plants; (**A**), phylogenetic tree showing the relative positions of 39 different gene encoding isoforms from higher plants, inferring by the maximum likelihood method of complete protein sequences; 10 sequences are P1-G6PDH, 10 sequences are P2-G6PDH, 8 sequences are P0-G6PDH; and 11 sequences are Cy-G6PDH. Legend for plant species: At, *Arabidopsis thaliana*; Os, *Oryza sativa Japonica Group*; Zm, *Zea mays*; Rc, *Rosa chinensis*; Vit, *Vitis vinifera*; Pp, *Prunus persica*; Solyc, *Solanum lycopersicum*; St, *Solanum tuberosum*. (**B**), List of G6PDH isoforms and their relative gene symbol from different higher plants, summarizing from the phylogenetic tree constructed in (**A**). The complete list of sequences using for tree construction is showed in Table S1.

In higher plants, G6PDHs reside in two cellular compartments, the cytosol and plastids [11,12]. Genome-wide analysis of Arabidopsis G6PDH indicates the existence of four plastidial (pla-G6PD) and two cytosolic (cy-G6PD) isoforms [13,14], also reported in several crops [15,16]. Plastidial G6PDH comprises three functional isoforms belonging to two distinct groups [P1 (G6PD1), P2 (G6PD2, G6PD3)] and a non-functional one (G6PD4) belonging to the P0 group. Cytoplasmic G6PDH is divided into two groups [P5 (G6PDH5) and P6 (G6PDH6)], providing 60–80% of the total activity. Besides their respective location, these G6PDH isoforms differ by their amino-sequences and their mode of regulation (Figure 2).

### 2.1. P1-G6PDH

The chloroplastic G6PDH has been defined as a P1-G6PDH and is mainly post-transcriptionally inhibited by high redox status (a high content of NADPH) and accumulation of ribulose-5P [17,18]. During photosynthetic electron transport in the light, a redox chain (the ferredoxin/thioredoxin system) inactivates the P1-G6PDH to guarantee an efficient photosynthesis, which activates the Calvin cycle and several stromal target enzymes such as fructose-1,6-bisphosphatase, NADP-malate dehydrogenase, phosphoribulokinase, and others [7,19]. This inhibitory effect of P1-G6PDH is removed in the dark when the NADPH level dropped, enabling the activation of the OPPP pathway to produce reducing equivalents [20]. 

### 2.2. P2-G6PDH

The plastidial P2-G6PDH is highly related to the rate of the OPPP, and expressed in almost all plant organs including growing tissues, photosynthetic, and non-photosynthetic tissues [13,21]. The activity of P2-G6PDH is more likely to be regulated in a similar manner as P1-G6PD, but their sensitivity to NADPH differs [22,23]. P2-G6PDH exhibits a 5–10 fold higher inhibition by NADPH than Cy-G6PDH and P1-G6PDH [24]. In addition, P2-G6PDH is much less sensitive than P1-G6PDH to regulation by thioredoxin and glutathione (GSH). A relatively significant role of P2-G6PDH generally consists in providing reductant in heterotrophic tissues in the absence of photochemical generation of reductants.

### 2.3. The Enigmatic P0-G6PDH

P0-G6PDH is considered as an enigmatic isoform found in peroxisomes and without any enzymatic activity. P1-G6PDH is involved in cysteine-dependent interaction with P0-G6PDH [25]. Due to the presence of a peroxisome targeting sequence (PTS) at the C-terminus of P0-G6PDH, these heterodimers can enter peroxisomes [26]. It is generally believed that the oxidative portion of the PPP exists in peroxisomes [27,28]. Additionally, some irreversible reactions of PPP are catalyzed by 6-phosphogluconolactonase (6-PGL) and 6-phosphogluconate dehydrogenase (6PGDH), both localized in the Arabidopsis peroxisome; hence, it is considered an efficient NADPH production mechanism in this organelle [26,29,30]. 

### 2.4. Cytosolic G6PDH (Cy-G6PDH)

In higher plants, the two cytosolic isoforms G6PD5 and G6PD6 are differently expressed in various tissues, even though they were initially purified from roots [13,21,31,32]. The cy-G6PD isoform is lowly sensitive to energy changes, which are regulated by the NADPH/NADP+ ratio and inhibited by NADPH [31]. However, the activity of Cy-G6PDH is regulated by a sugar-sensing mechanism, and plays an important role in amino acid biosynthesis according to the carbon status of plants [33]. Moreover, Cy-G6PDH expression is tightly induced by abscisic acid (ABA; Hou et al., 2006) and sensitive to light [34], which mainly control the activity of P1-G6PDH [35].

## 3. G6PDH and Seed Germination

In the 1980s, a great deal of studies carried out on several species (e.g., *Gossypium*, *Pisum sativum*, *Arachis hypogaea*, *Prunus cerasus*, *Phaseolus mungo*, *Avena fatua*) reported a tight correlation between dormancy breaking treatments and increased G6PDH activity. These investigations were based on the activity of the two cytosolic enzymes of the OPPP (G6PDH and 6-PDGH) and one glycolysis-related enzyme (aldolase) in seed tissues during dormancy-breaking treatments (e.g., stratification, after-ripening). Aldolase catalyzes the conversion of fructose 1-6-diphosphate to glyceraldehyde 3-phosphate and dihydroxy-acetone phosphate through the glycolysis pathway [36]. These authors hypothesized that activation of the OPPP in germinating seeds played a pivotal role in dormancy breaking and storage mobilization by controlling redox homeostasis and enzyme activities [37,38,39,40,41,42,43]. The OPPP was found highly induced during seed germination, much more so in the endosperm than in the radicle [44]. The specificity of the spatial regulation of the OPPP pointed out the respective roles of these sub-compartments in the regulation of Arabidopsis seed germination.

**Figure 3:**
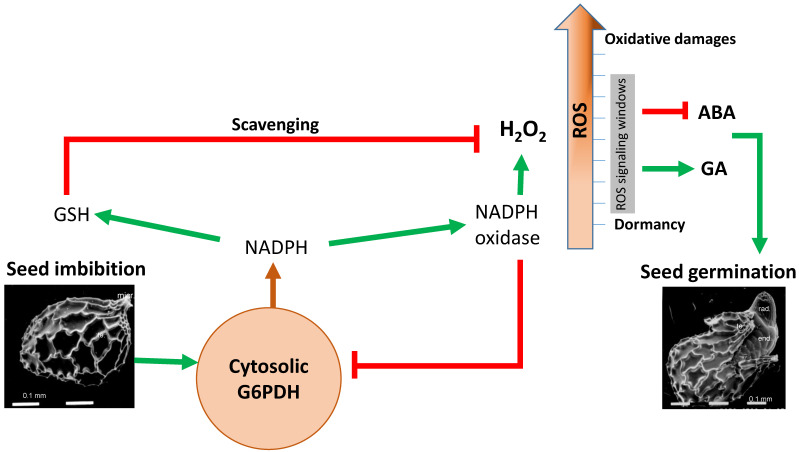
Cytosolic G6PDH in radicle of imbibed seed modulates ROS homeostasis and hormonal signaling in the control of seed germination. The NADPH provided by cytosolic G6PDH activity is required both for ROS production by NADPH oxidase and for ROS scavenging by activation of GSH ascorbate cycle; the working model based on genetic evidence proposed that G6PDH modulates ROS to a steady state level controlling ABA and GA activities and dormancy release; GSH, reduced glutathione; ROS, reactive oxygen species; ABA, abscisic acid; GA, gibberellic acid.

The involvement of different isoforms of G6PDH—cytosolic as well as plastidial ones—in dormancy release was investigated. It was proposed that Cy-G6PDH controls germination by maintaining a steady-state level of ROS (Figure 3) required for breaking dormancy [45,46], yet a limited one to avoid excessive oxidative damage in the root apical meristems [47]. Genetic evidence supports that ROS provided by NADPH oxidase in germinating seeds under salt stress stimulate the two cytosolic G6PDHs (Cy-G6PDH5 and Cy-G6PDH6) and increase the cytosolic NADPH content, which in turn dampens ROS damage by activating the GSH–ascorbate cycle involved in H_2_O_2_ scavenging [48]. Using *cy-g6pdh* deficient mutants, researchers demonstrated that Cy-G6PD5 is required to release dormancy and reduce seed sensitivity to ABA—a hormone involved in germination inhibition—through the repression of the *Abscisic Acid Insensitive 5* (*ABI5*) gene [49]. The authors described that ABA induces excessive accumulation of ROS in germinating seeds and seedlings of the cy-*g6pd5*-deficient mutant. ROS control dormancy through post-transcriptional regulation by selective proteins and via RNA oxidation [50,51], cell wall loosening for cell wall elongation, and endosperm weakening [52], the response to ethylene, or through the control of the ABA catabolism/GA biosynthesis [53,54]. Nevertheless, to avoid the deleterious effects of ROS, their level must be finely balanced by the reducing power or detoxifying enzymes that seem to be largely provided by OPPP-derived NADPH. Moreover, Cy-G6PD is described to be induced by ABA, hydrogen peroxide (H_2_O_2_), or nitric oxide (NO) [15,49,55] and is crucial for plant tolerance to stresses, more likely by maintaining the redox balance [33]. Controlling the redox status of the germinating seeds should be central to optimize plant fitness. At this developmental stage, cyt-G6PDH may modulate both the adaptive mechanisms of dormancy and the plant responses to biotic and abiotic stresses.

The contribution of G6PDH in reserve mobilization through the regulation of thioredoxin (Trx) at the end of germination has also been suggested. Many investigations have underlined the important role of NADPH-dependent Trx systems in reserve mobilization [56,57,58,59,60,61]. Cytosolic Trx-dependent reduction in storage proteins provides essential organic resources for the transition of germinating seeds to autotrophic seedlings. Moreover, seed plastidial y-type Trxs (Trx y) induce plastidial P1-G6PDH, a major source of reducing power in heterotrophic tissues [62]. A functional genetic approach documented that y-type Trxs contribute to seed germination by regulating ROS levels through the activation of plastidial G6PDH [37,63].

Elucidating the molecular mechanisms whereby G6PDH controls the hormonal metabolism and hormone sensing, ROS detoxification, but also the carbon metabolism and carbon reallocation during the germinating-seed-to-autotrophic-seedling transition will be crucial in the near future (Figure 3). A recent analysis of the whole transcriptome changes following nitrate treatment during seed imbibition showed that genes involved in nitrate assimilation and transport as well as the plastidial P2-G6PDH were upregulated, highlighting a potential link between G6PDH and the well-known nitrate signaling effect on the ABA catabolism and dormancy release.

## 4. G6PDHs and Nitrogen Assimilation

Nitrogen (N) is one of the most limiting factors for plant growth and productivity. Consequently, plants have various mechanisms for maximum N efficiency [64]. Plant nitrogen nutrition occurs through organic (amino acids, urea) and inorganic (nitrate, ammonium) forms of nitrogen and is governed by a complex regulatory network [65,66]. The main route of nitrate assimilation involves nitrate reductase (NR) and nitrite reductase (NiR), resulting in its reduction into ammonium (NH_4_), which is incorporated into amino acids through the joint action of glutamine synthetase (GS) and the glutamine oxoglutarate aminotransferase (GOGAT) cycle [67,68,69]. A link between the OPPP and inorganic nitrogen assimilation has mainly been reported in non-photosynthetic tissues (e.g., root systems), with a prevailing role of plastidial G6PDH [19], which can meet the high demand for reducing power upon nitrogen assimilation [3]. Early data showed that NiR activity in barley roots relies on elevated levels of NADPH, suggesting that G6PDH could play a key role during nitrate assimilation [70]. In accordance with this, synthesis of glutamate (a final product of the GOGAT cycle) by isolated pea root plastids fed with two GOGAT substrates (glutamine and 2-oxoglutarate) requires the OPPP substrates (Glc6P, ribose P), coordinated with the reducing activity of G6PDH [70,71]. Exogenous supply of inorganic nitrogen (nitrate or ammonium) to barley roots induced plastidial G6PDH at both the transcriptional and protein levels [21,72]. This nitrate-dependent upregulation of G6PDH was also reported in seedlings of NIR knockout mutants, assuming that it is directly triggered by the nitrate-related signaling pathway [73,74]. This fine tuning between nitrogen and the OPPP was transposed to the induction of the major root nitrate uptake transporters (NRT1.1 and NRT2.2) in Arabidopsis roots [75,76]. In this case, photosynthesis-derived sugar induces nitrate transporters in the roots, which are repressed by 6-amino-nicotinamide (6-AN), an inhibitor of both G6PDH and 6PGDH (6-phospho-gluco-dehydrogenase) [77,78]. This effect is independent of the hexokinase- and trehalose-6P signaling pathway [75], supporting that sugar-dependent upregulation of nitrate transporters involves an OPPP-dependent signaling mechanism. Taken together, the OPPP and nitrogen uptake/assimilation by heterotrophic organs (roots) are a tightly coordinated process [79], with a key role of root plastidial G6PDH [3]. Huge efforts are still required to disclose the molecular regulatory network mechanisms governing this coordination between nitrogen assimilation and G6PDH regulation. Early studies indicate that the promoter sequences of NiR and G6PDH present the same NIT2 motif, which is a N-metabolism regulating factor [80,81]. Based on this, a promising line of study would be to investigate this close relationship between the OPPP and nitrogen assimilation in other non-photosynthetic organs such as vegetative buds, which also require organic and inorganic nitrogen to sustain their outgrowing activity [65]. Such a study would obviously bring new insights into the common and specific mechanisms in different biological contexts.

## 5. G6PDHs and Plant Branching

Shoot branching is crucial for plant development and yield and is greatly dependent on the ability of axillary buds to grow out along the stem [82,83,84]. Bud outgrowth is very finely regulated by multiple endogenous (e.g., hormones, sugar) and exogenous (e.g., light, water stress) cues [85,86,87,88,89,90,91,92]. In this intricate regulation, sugars behave as signaling entities that promote bud outgrowth through several sugar signaling pathways corresponding to the trehalose 6P-, hexokinase-, glycolysis/tricarboxylic acid (TCA)-, and OPPP-dependent signaling pathways [83,93,94]. The involvement of the OPPP in plant branching was identified following the discovery that bud outgrowth relies on the bud H_2_O_2_ content: cytokinins (CKs) promote bud outgrowth by reducing H_2_O_2_ through the induction of the GSH/ascorbate cycle, and, conversely, H_2_O_2_ accumulation reduces bud ability to grow out [95,96]. The authors of [94] first evidenced the role of the OPPP in sugar branching by demonstrating that the promotive effect of sucrose on bud outgrowth is repressed by 6-AN—an inhibitor of G6PDH—in *Rosa* sp. Molecular experiments conducted on in vitro-cultured vegetative buds and on stably transformed *Rosa* calluses revealed that the OPPP-dependent signaling pathway is involved in both sugar-mediated transcriptional (promoter level) and posttranscriptional (3′untranslated region) downregulation of Teosinte Branched 1/Branched1 (TB1/BRC1) [91,94,97]. BRC1 is the main inhibitor of shoot branching [98]. An OPPP-specific 300-bp region was identified in the *BRC1* promoter between 1900 and 1600 bp [94]; its 3′UTR contains six putative motifs of the Pumilio/FBF RNA-binding protein family (PUF) [97]. One future task will consist of deciphering the molecular mechanisms underlying the OPPP-mediated downregulation of *BRC1*.

## 6. G6PDHs and Sugar Signaling

Sugar perception and signaling enable plants to integrate various internal and external cues to achieve nutrient homeostasis, mediate developmental programs, and orchestrate their stress response [99,100]. To sense different sugars, plants have evolved a complex mechanistic system that includes hexose-, disaccharide-, and the OPPP–signaling pathways [101,102]. The OPPP–dependent signaling pathway has been reported in two biological contexts related to sink organs. It drives sugar-mediated stimulation of nitrogen and sulfur acquisition by the roots, downstream and independently of hexokinase signaling and the trehalose-6P signaling pathway [75,76]. It has also been described as a main signaling route of sugar-dependent stimulation of bud outgrowth [94]. However, OPPP-dependent sugar signaling might be more complex and operate through different pathways [75,79]. Three hypotheses are still plausible: (1) one of the carbon metabolites generated through the OPPP could act as a cue; (2) an enzyme of the OPPP—e.g., G6PDH—could exhibit a dual (catalytic and signaling) function like hexokinase (HXK) does [103]; and (3) NADPH resulting from G6PDH activity could be involved in redox regulation via the NADPH-dependent signaling pathway. This latter hypothesis was recently reported for the sugar-mediated regulation of *NRT2.1* expression in the roots, which is related to the redox status of the plant [104]. This regulatory mechanism might also be involved in the OPPP-mediated sugar stimulation of bud outgrowth. Recent data indicate that the ability of *Rosa* buds to grow out depends on their redox status: high levels of H_2_O_2_ in the buds strongly prevent outgrowth, while high activity of the GSH–ascorbate cycle, which is directly linked to the activity of G6PDH, decreases the H_2_O_2_ content and promotes bud outgrowth [95]. The relevance of the GSH–ascorbate cycle has been confirmed for CK-induced bud outgrowth under darkness [96]. In this case, CKs positively affect GSH synthesis to stimulate H_2_O_2_ scavenging, and this allows for bud outgrowth [96]. However, whether CKs regulate G6PDH inside the buds still remains to be unveiled, while we do know that G6PDH is upregulated by sugar [90]. It will be of great interest to deeply investigate the role of G6PDH in bud outgrowth regulation by sugars, hormones, and ROS, and to identify the OPPP-dependent regulatory molecular network. All these findings lay the cornerstones for deciphering the exact role of G6PDH in shoot branching, for instance, by characterizing the branching phenotype of G6PDH-deficient Arabidopsis mutants.

## 7. G6PDH and Abiotic Stress

### 7.1. Identification of Link between G6PDH and Abiotic Stress

As sessile organisms, plants have to cope with various abiotic stresses such as salinity, drought, or temperature changes [105]. G6PDHs play a crucial function in modulating redox homeostasis when plants are exposed to abiotic stresses [3,106,107,108]. Some researchers found the role of a cytosolic G6PDH (*NbG6PDH-Cyto*) and two plastidial isoforms of G6PDH (*NbG6PDH-P1* and *NbG6PDH-P2*) in the stress physiology of *Nicotiana benthamiana* [109]. ROS production in link with hypersensitive-response (HR) cell death and NADPH oxidase (NOX, also known as respiratory burst oxidase, RBOH) activity decreased in *NbG6PDH-P2-silenced* plants. Silencing of the cytosolic NAD kinase NbNADK1, which phosphorylates NADH to NADPH, compromised HR cell death and ROS production. The concomitant silencing of NbG6PDH-P2 reduced HR cell death and ROS down to levels near those of NbG6PDH-P2-silenced plants. These authors suggested that NADPH produced by *NbG6PDH-P2* was responsible for HR cell death and ROS production mediated by RBOH. Increased G6PDH activity could also stimulate ROS generation by regulating NADPH oxidase activity in rice [110]. G6PDH may be involved in maintaining redox homeostasis by regulating the activity of superoxide dismutase (SOD), peroxidase (POD), and ascorbate peroxidase (APX) [111,112,113]. Treatment with Acibenzolar-S-methyl (an inducer of disease resistance in plants) increased G6PDH activity and the ascorbic acid (AsA) level, while the GSH and NADPH contents and the expression level of redox homeostasis-related genes such as SOD, APX, or dehydroascorbate reductase (DHAR) were reduced [114]. G6PDH activity markedly increased in soybean under drought stress [115]. Upon PEG6000 treatment, the activity of the antioxidant enzymatic machinery (superoxide dismutase (SOD), catalase (CAT), peroxidase (POD), glutathione reductase (GR), dehydroascorbate reductase (DHAR), monodehydroascorbate reductase (MDHAR)) increased in soybean, and GSH and ascorbic acid (AsA) reached elevated levels; once again, these results demonstrate that G6PDH plays a central role in redox homeostasis by maintaining the GSH and Asc levels.

### 7.2. Saline-Alkaline Stress and Aluminum Toxicity

Saline-alkaline stress is one of the most serious global issues in plant production [116,117,118]. The metabolic changes triggered by salt stress result in a high need for reductants supplied by the OPPP, consistently with increased total G6PDH activity [119]. *Arabidopsis thaliana* glycogen synthase kinase3 (ASKα) regulates stress tolerance by activating G6PDH, which is essential for maintaining the cellular redox balance. Loss of stress-activated ASKα impairs G6PDH activity, increases ROS levels, and enhances sensitivity to salt stress, while ASKα-overexpressing plants exhibit high G6PDH activity, lower ROS levels and are more tolerant to salt stress [33]. In wheat (*Triticum aestivum* L.), *G6PDH* transcripts were rapidly induced at the early stage of NaCl treatment (almost a 2.2-fold increase), indicating that G6PDH is involved in the initial response of plants to salt stress [120]. In highland barley (*Hordeum vulgare* var. nudum Hook. f.), cy-G6PDH confers resistance to alkaline stress and ultimately improves fresh weight and photosynthetic activity through NADPH production and accumulation of reduced GSH [121]. In line with these results, some researchers cloned five G6PDH genes (*HvG6PDH1* to *HvG6PDH5*) from highland barley and characterized their respective roles in the response to abiotic stresses. The analysis of enzyme activities and gene expression showed that *HvG6PDH1* to *HvG6PDH4* were involved in the responses to salt and drought stresses [122]. Cytosolic *HvG6PDH2* is the major isoform against oxidative stress. *HvG6PDH1* to *HvG6PDH4* and their encoded enzymes responded to jasmonic acid (JA) and ABA treatments, implying that JA and ABA are probably key regulators of HvG6PDHs [122,123,124]. Some researchers characterized the root behavior of two Arabidopsis single null mutants (*g6pdh5* and *g6pdh6*), one double mutant (*g6pdh5*/*6*), and two cy-G6PDH isoforms to salt stress exposure. The seed mutants displayed a reduced germination rate, reduced root elongation, and high accumulation of ROS under salt stress compared to the wild type [48]. Interestingly, the alteration of *G6PDH5* and *G6PDH6* expression affected the activities and transcript levels of various antioxidant enzymes, especially APX and GR. Exogenous application of ascorbic acid and GSH rescued the seed and root phenotypes of *g6pdh5/6*. In response to salt stress, some researchers characterized the nine members of the *G6PDH* gene family (*GmG6PDHs*) in soybean [125]. The activities and transcripts of *GmG6PDHs* were dramatically stimulated, with a notable role of *GmG6PDH2*—a cytosolic isoform. Enzymatic assays of recombinant GmG6PDH2 proteins expressed in *Escherichia coli* (*E. coli*) showed that this enzyme has functional NADP^+^-dependent G6PDH activity, and *GmG6PDH2*-overexpressing plants exhibited a high degree of resistance to salt stress related to a close coordination of the redox state, the ascorbic acid pool and the GSH pool. The G6PDH activity was enhanced rapidly in the presence of 100mM NaCl in *Phaseolus vulgaris*, which is associated with a raise of G6PDH protein [126]. Application of a G6PDH inhibitor blocked the increase in G6PDH and nitrate reductase activity, as well as NO production. Therefore, G6PDH plays a pivotal role in nitrate-reductase-dependent NO production and in tolerance to salt stress.

Besides salt stress, exposure to high aluminum concentrations significantly induced total and cytosolic G6PDH activities in soybean roots, along with NO accumulation [127]. NADPH produced by NO-modulated cytosolic G6PDH is responsible for ROS accumulation mediated by NADPH oxidase under aluminum stress. Further investigations using pharmacological and transgenic approaches demonstrated that G6PDH positively regulates the activity of NADPH oxidase under aluminum treatment. These results suggest that G6PDH mediates Al-induced programmed cell death through NADPH oxidase-dependent ROS production [128].

### 7.3. Drought and Heat

Drought can significantly increase the enzymatic activities of cytosolic G6PD (Cyt-G6PD) and plastidial G6PD (P2-G6PD) possibly triggered by NO and H_2_O_2_ in soybean roots [55]. In winter wheat, *TaG6PDH* (*Triticum aestivum G6PDH*) expression was upregulated under cold stress and exogenous ABA application, suggesting that TaG6PDH positively responds to cold stress and ABA [129]. Similarly, *FaG6PDH* positively regulated cold tolerance in strawberry [130], and in silico bioinformatics analysis of 19 *FaG6PDH* promoters revealed the presence of at least one stress-responsive cis-acting element [131]. An early stress response would involve the OPPP, which represents a true metabolic sensor during the response to various stresses. These results indicate that *G6PDH* might play a pivotal role in redox homeostasis, ROS signaling, and NO cascade signaling. In line with this statement, the activity of G6PDH and antioxidant enzymes (APX, CAT, POD, and SOD) in *Przewalskia tangutica* and tobacco (*Nicotiana tabacum* L.) calluses increased after 40 °C treatment. When G6PDH was partially inhibited by glucosamine pretreatment, the antioxidant enzyme activities, the H_2_O_2_ content and plasma membrane NADPH oxidase activity decreased, while H_2_O_2_ application increased the activity of G6PDH and antioxidant enzymes [132]. In *Phaseolus vulgaris*, the heat-sensitive genotype had a higher G6PDH activity than the control under normal temperature [133]. However, under elevated temperature treatment, G6PDH activity increased by approximately 78% in the heat-insensitive genotype but decreased by approximately 37% in the sensitive genotype. These results indicate that G6PDH confers plants heat stress tolerance by regulating H_2_O_2_ levels under heat stress. Tomato plants grown for 30 days and 45 days without irrigation exhibited 1.67- and 1.32-fold higher total G6PDH activity, respectively [10]. 

**Figure 4:**
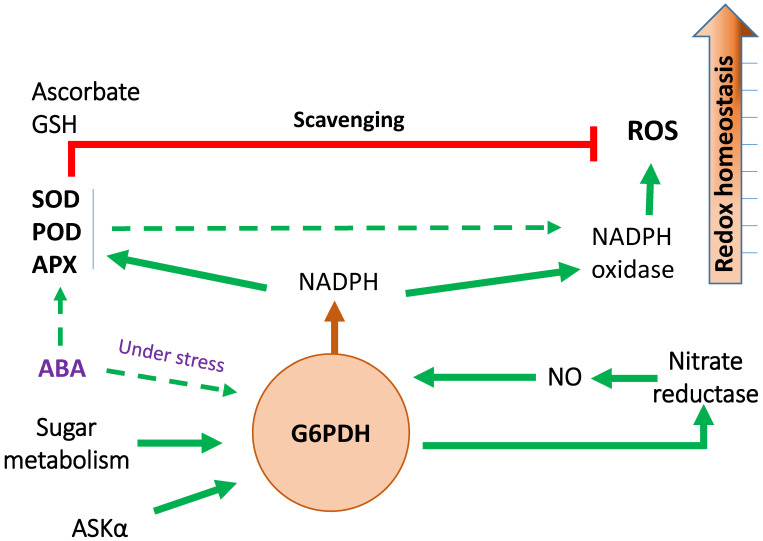
Regulatory network of G6PDH in the context of plant responses to abiotic stress. The NADPH provided by cytosolic G6PDH activity is required both for ROS production by NADPH oxidase and for ROS scavenging by activation of GSH ascorbate cycle. The working model proposed that G6PDH plays a pivotal role in redox homeostasis, ROS signaling and NO cascade signaling. ASKα, *Arabidopsis thaliana* glycogen synthase kinase3 (GSK3)/SHAGGY-like kinase; ABA, abscisic acid; APX, ascorbate peroxidase; POD, peroxidase; SOD, superoxide dismutase; GSH, reduced glutathione; ROS, reactive oxygen species.

Although all these findings clearly underline the crucial role of G6PDH in plant tolerance to abiotic stress (Figure 4), many efforts should be deployed to provide a comprehensive picture of the molecular regulatory network governing G6PDH regulation in the context of plant responses to harmful conditions. One outstanding question will be to elucidate whether these different abiotic stresses mediate upregulation of G6PDH through common or specific regulatory pathways and to decipher how G6PDH is regulated in response to multiple abiotic stresses.

## 8. Conclusions

Glucose-6-phosphate dehydrogenases (G6PDHs) are cytosolic or plastidial enzymes that catalyze the oxidation of glucose-6-phosphate (G6P) to 6-phosphogluconolactone. Their activity diverts part of G6P from glycolysis to the oxidative pentose phosphate pathway (OPPP), so that it is key for determining the balance between these two metabolic pathways. As the OPPP is also a critical producer of the reductant component NADPH, G6PDHs play a determining role in ROS scavenging. Besides their role in physiological processes, G6PDHs play a role in plant life cycle and development, especially by influencing seeds (germination) and axillary bud dormancy (plant branching), nitrogen assimilation, responses to abiotic stresses, and by contributing to the recently identified sugar signaling pathway. Further studies are required to (1) understand the respective roles of the different G6PDH isoforms and their regulation, (2) decipher the molecular mechanisms allowing G6PDHs to influence the development and the responses of plants to their environment, and (3) unveil the role of ROS regulation in these processes. An additional interesting research question would be to better understand the interaction between G6PDHs and the molecular regulatory network of hormones and nutrients.

## Data Availability

Not applicable.

## Supplementary Materials

The following supporting information can be downloaded at: https://www.mdpi.com/article/10.3390/ijms232416128/s1.
