# Supplementary material for: Glucose-6-Phosphate Dehydrogenases: The Hidden Players of Plant Physiology
Source: Int J Mol Sci. 2022 Dec 17;23(24):16128. doi: 10.3390/ijms232416128 (PMC9785579; doi:10.3390/ijms232416128)
Supplement: Supplementary file 1 [file ijms-23-16128-s001.zip › ijms-2085444-supplementary.pdf]

Supplementary data

Table S1. List of relative gene information encoding different G6PDH isforms in 8 higher plants.

**Gene symbol**	**Organism**	**Protein accession**	**Gene stable ID**
G6PD1	*Arabidopsis thaliana*	NP_198428.1	AT5G35790
G6PD2	*Arabidopsis thaliana*	NP_196815.1	AT5G13110
G6PD3	*Arabidopsis thaliana*	NP_173838.1	AT1G24280
G6PD4	*Arabidopsis thaliana*	NP_563844.1	AT1G09420
G6PD5	*Arabidopsis thaliana*	NP_189366.1	AT3G27300
G6PD6	*Arabidopsis thaliana*	NP_198892.1	AT5G40760
LOC4329889	*Oryza sativa Japonica Group*	XP_015627059.1	Os02g0600400
LOC4336209	*Oryza sativa Japonica Group*	XP_015635836.1	Os04g0485300
LOC4342988	*Oryza sativa Japonica Group*	XP_015647088.1	Os07g0406300
LOC4333103	*Oryza sativa Japonica Group*	XP_015628664.1	Os03g0412800
LOC4332659	*Oryza sativa Japonica Group*	XP_025879520.1	Os03g0318500
LOC112187819	*Rosa chinensis*	XP_024182532.1	RchiOBHm_Chr2g0156161
LOC112200384	*Rosa chinensis*	XP_024197181.1	RchiOBHm_Chr4g0429721
LOC112187722	*Rosa chinensis*	XP_024182385.1	RchiOBHm_Chr2g0155901
LOC112193013	*Rosa chinensis*	XP_024188781.1	RchiOBHm_Chr3g0457961
LOC112185338	*Rosa chinensis*	XP_024179344.1	RchiOBHm_Chr2g0174311
LOC112175829	*Rosa chinensis*	XP_024169347.1	Nd
LOC101258470	*Solanum lycopersicum*	XP_004243567.1	Solyc07g045540
LOC101253589	*Solanum lycopersicum*	XP_004239245.1	Solyc05g015950
LOC101258494	*Solanum lycopersicum*	XP_004230338.1	Solyc01g100960
LOC101249507	*Solanum lycopersicum*	XP_004231802.1	Solyc02g093830
LOC102594145	*Solanum tuberosum*	NP_001275038.1	X83923
LOC102597484	*Solanum tuberosum*	NP_001275072.1	CAB52708
LOC102592547	*Solanum tuberosum*	XP_006344803.1	Nd
LOC102595038	*Solanum tuberosum*	NP_001275397.1	X74421
LOC100284317	*Zea mays*	NP_001150684.1	GRMZM2G130230
LOC100279290	*Zea mays*	NP_001352992.1	GRMZM2G177077
LOC100383421	*Zea mays*	NP_001169544.1	GRMZM2G031107
LOC100304292	*Zea mays*	NP_001348206.1	GRMZM2G426964
LOC100383765	*Zea mays*	NP_001169871.1	GRMZM2G179521
LOC18790433	*Prunus persica*	XP_007226936.1	PRUPE_1G222100
LOC18781823	*Prunus persica*	XP_020415160.1	PRUPE_3G198600
LOC18772820	*Prunus persica*	XP_007208319.1	PRUPE_6G307600
LOC18783720	*Prunus persica*	XP_007215121.1	PRUPE_3G300200
LOC100264918	*Vitis vinifera*	XP_002268887.1	Nd
LOC100267681	*Vitis vinifera*	XP_002266930.1	VIT_00000913001
LOC100254628	*Vitis vinifera*	XP_010659726.1	VIT_00031295001
LOC100254253	*Vitis vinifera*	XP_002266527.1	VIT_00001847001
LOC100266583	*Vitis vinifera*	XP_010657480.1	VIT_00030086001

All information is based on NCBI website (https://www.ncbi.nlm.nih.gov), Nd means

lack of relevant information.
